# Calmodulin Regulates Human Ether à Go-Go 1 (hEAG1) Potassium Channels through Interactions of the Eag Domain with the Cyclic Nucleotide Binding Homology Domain*

**DOI:** 10.1074/jbc.M116.733576

**Published:** 2016-06-20

**Authors:** Eva Lörinczi, Matthew Helliwell, Alina Finch, Phillip J. Stansfeld, Noel W. Davies, Martyn Mahaut-Smith, Frederick W. Muskett, John S. Mitcheson

**Affiliations:** From the ‡ Department of Molecular and Cell Biology, University of Leicester, Leicester LE1 9HN,; the § School of Physiology and Pharmacology, University of Bristol, Bristol BS5 1TD, and; the ¶ Department of Biochemistry, University of Oxford, Oxford OX1 3QU, United Kingdom

**Keywords:** calcium imaging, calmodulin (CaM), electrophysiology, gating, potassium channel

## Abstract

The ether à go-go family of voltage-gated potassium channels is structurally distinct. The N terminus contains an eag domain (eagD) that contains a Per-Arnt-Sim (PAS) domain that is preceded by a conserved sequence of 25–27 amino acids known as the PAS-cap. The C terminus contains a region with homology to cyclic nucleotide binding domains (cNBHD), which is directly linked to the channel pore. The human EAG1 (hEAG1) channel is remarkably sensitive to inhibition by intracellular calcium (Ca^2+^*_i_*) through binding of Ca^2+^-calmodulin to three sites adjacent to the eagD and cNBHD. Here, we show that the eagD and cNBHD interact to modulate Ca^2+^-calmodulin as well as voltage-dependent gating. Sustained elevation of Ca^2+^*_i_* resulted in an initial profound inhibition of hEAG1 currents, which was followed by a phase when current amplitudes partially recovered, but activation gating was slowed and shifted to depolarized potentials. Deletion of either the eagD or cNBHD abolished the inhibition by Ca^2+^*_i_*. However, deletion of just the PAS-cap resulted in a >15-fold potentiation in response to elevated Ca^2+^*_i_*. Mutations of residues at the interface between the eagD and cNBHD have been linked to human cancer. Glu-600 on the cNBHD, when substituted with residues with a larger volume, resulted in hEAG1 currents that were profoundly potentiated by Ca^2+^*_i_* in a manner similar to the ΔPAS-cap mutant. These findings provide the first evidence that eagD and cNBHD interactions are regulating Ca^2+^-dependent gating and indicate that the binding of the PAS-cap with the cNBHD is required for the closure of the channels upon CaM binding.

## Introduction

The ether à go-go potassium channel family (KCNH)^2^ of voltage-gated potassium channels consists of three subgroups, ether à go-go (EAG), EAG-related gene (ERG), and EAG-like potassium (ELK) channels. In recent years, most of the focus has been on the ERG subfamily because of their crucial role in cardiac repolarization (1). Relatively less is known about the physiological role of EAG channels. In *Drosophila*, where EAG channels were first discovered, the behavioral mutant (eag) causes spontaneous repetitive action potential (AP) firing in motor neurons and increased transmitter release that results in flight muscle paralysis (2). In mammals, EAG channel expression is normally restricted to the central nervous system, particularly the hippocampus, cerebellum, and brain stem (3). Recently, a role for EAG channels in regulating presynaptic calcium and neurotransmitter release during high frequency trains of APs has been demonstrated in mouse cerebellar synapses (4). hEAG1 and hEAG2 channels are also aberrantly overexpressed in human cancers. hEAG1 is highly expressed in >75% of non-CNS cancers (5–8) and hEAG2 in a substantial subset of patients with medulloblastomas (9).

hEAG1 channels are exquisitely sensitive to [Ca^2+^]*_i_*, with a half-maximal inhibition at ∼100 nm (10, 11). Regulation of ion channels by Ca^2+^*_i_* is critical for converting Ca^2+^ signals into electrical signals (*e.g.* slow and fast after hyperpolarizations), for altering the balance of ionic currents during APs, and for modulating membrane excitability. It is likely that Ca^2+^*_i_* regulation of EAG1 channels exerts a feedback function that is important for long term effects in neuronal signaling (10–13). Because Ca^2+^ signaling is also important during the cell cycle, Ca^2+^*_i_* regulation of hEAG1 channels may also be functionally important in cell proliferation and cancer progression (14).

**FIGURE 1.**
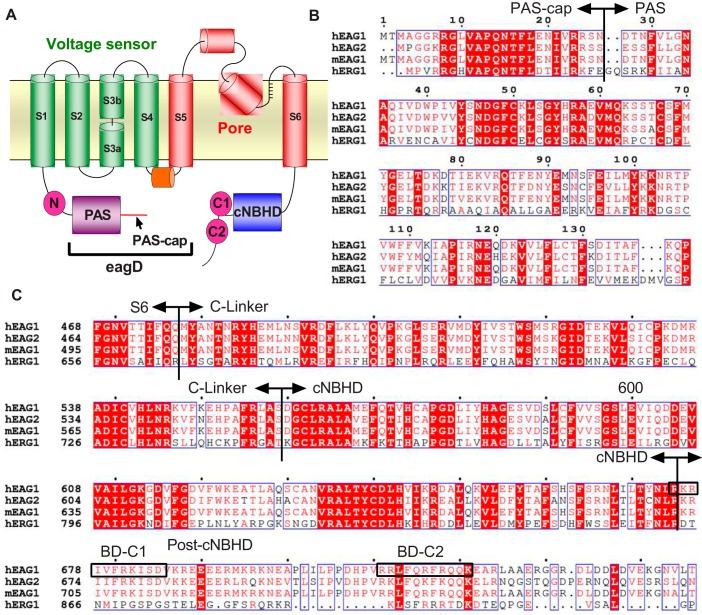
**hEAG1 secondary structure and sequence alignments.** *A,* schematic representation of the secondary structure of hEAG1 K^+^ channels showing the location of three CaM binding domains (*magenta circles*) per subunit. CaM binding domain N (*BD-N*) is on the N terminus, close to the PAS domain. CaM binding domains C1 and C2 (*BD-C1* and *BD-C2*) are located on the C terminus and adjacent to the cNBHD. *B* and *C,* protein sequence alignments of hEAG1 eagD (*B*) and cNBHD (*C*) with other KCNH family members. Numbering refers to hEAG1 sequences. *White text* on a *red background* indicates identical sequence, and *red text* indicates a semi-conserved sequence. *Black boxes* indicate positions of BD-C1 and BD-C2 in the post-cNBHD sequence of hEAG1.

Like other voltage-gated K^+^ channels, the central pore of KCNH channels is formed by the tetrameric assembly of S5–S6 helices and is surrounded by voltage sensor domains formed by S1–S4. The N terminus of hEAG1 contains an eag domain (eagD), which is unique to the KCNH channel family and contains a Per-Arnt-Sim (PAS) homology domain. PAS domains are structural folds that mediate protein-protein interactions in a variety of signaling proteins (15). In KCNH channels, the PAS domain is preceded by a highly conserved sequence of 25–27 amino acids that has become known as the PAS-cap (see Fig. 1) (16). NMR studies reveal that the first part of the PAS-cap is disordered, whereas the second half contains a stable amphipathic α-helix (17–19). Both segments have been shown to be important for gating of hEAG1 and hERG1 channels (17, 18, 20–22). The C terminus of the KCNH channel family contains a cyclic nucleotide binding homology domain (cNBHD) that is structurally similar to the cyclic nucleotide binding domains of CNG and HCN channels (23–26). However, KCNH channels lack key residues for cyclic nucleotide binding and are not directly regulated by cAMP or cGMP (27). Instead, the functional role of the KCNH cNBHD appears to be to regulate channel gating through interactions with the eagD (17, 28–30). The cNBHD is connected to the S6 inner helix of the pore by a region of ∼60 amino acids known as the C-linker, providing a mechanism for coupling conformational changes in the cNBHD to changes in gating of the pore (Fig. 1).

The mechanism and structural basis for regulation of hEAG1 channels by Ca^2+^*_i_* are largely unknown. Elegant studies by Schonherr *et al.* (12) and Ziechner *et al.* (13) demonstrated that calmodulin (CaM) is the Ca^2+^*_i_* sensor and inhibits hEAG1 currents by binding to the channels in a Ca^2+^-dependent manner. Three CaM binding domains (BD) have been identified using *in vitro* assays; two are on the C terminus close to the cNBHD (BD-C1, 674–683, and BD-C2, 711–721) and one is on the N terminus close to the eagD (BD-N, 151–165). GST fusion proteins containing BD-N or BD-C2 bind CaM in a strong Ca^2+^-dependent manner with dissociation constants in the nanomolar range (13). F151N/L154N mutations of BD-N and F714S/F717S mutations of BD-C2 reduce the *K_d_* of Ca^2+^-CaM by ∼20- and 6-fold, respectively, in isolated channel fragments and also substantially reduce the CaM-dependent inhibition of functional channels in excised patches (12, 13). The binding affinity of BD-C1 for Ca^2+^-CaM seems to be much weaker, but nevertheless, biochemical and functional studies suggest it plays a role, perhaps as part of a complex involving BD-C2 (12, 13, 26, 31).

Analysis of the secondary structure of hEAG1 channels reveals that all the CaM-binding sites are located adjacent to the eagD and cNBHD. Given the importance of these structural domains for regulating voltage-dependent gating, we hypothesized that interactions between the eagD and cNBHD could also be critical for Ca^2+^-CaM-dependent regulation of hEAG1. In this study we show that the Ca^2+^-CaM-dependent regulation is more complex than previously described. Elevated Ca^2+^*_i_* results in an initial profound inhibition, which is followed by a second phase not previously reported, during which current amplitudes begin to recover, although activation gating is profoundly slowed and shifted to depolarized potentials. Deleting either the eag domain or cNBHD completely abolishes calcium sensitivity. Intriguingly, deletion of just the PAS-cap (residues 2–26) results in a paradoxical increase of current in response to elevated intracellular calcium, which is mimicked by specific cNBHD mutations. Collectively, these results demonstrate a novel role for the eagD and cNBHD in coupling Ca^2+^-CaM binding to closure of the channel pore. Furthermore, they suggest that hEAG1 channels undergo multiple conformational changes in response to Ca^2+^-CaM binding and that the PAS-cap is required to stabilize the closed conformation.

## Results and Discussion

### 

#### 

##### Wild-type hEAG1 Channels Undergo Two Distinct Phases of Inhibition in Response to Elevated Ca^2+^_i_

**FIGURE 2.**
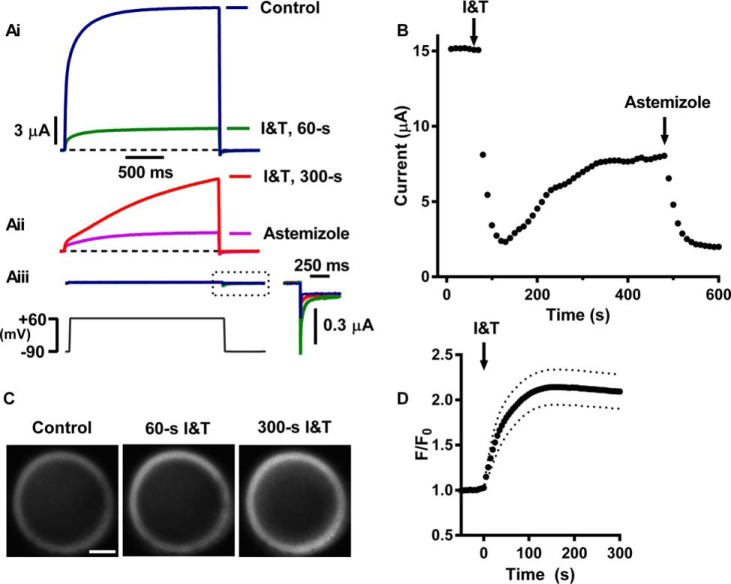
**Wild-type hEAG1 channels are profoundly inhibited by raising cytoplasmic calcium.** *A,* representative recordings of WT hEAG1 currents recorded with voltage steps to +60 mV from a holding potential of −90 mV, before (control) and during application of 5 μm of both ionomycin and thapsigargin (*I & T*). I and T resulted in a profound initial inhibition (*Ai, green trace* recorded 60 s after I and T application). In most cells, sustained I and T application resulted in the development of a slowly activating current (*Aii, red trace*) that progressively increased in amplitude and was inhibited by 50 μm astemizole (*Aii, magenta trace*). *Aiii,* representative current traces from an oocyte injected with diethyl pyrocarbonate water recorded in control solution (*blue*) or after 60 s (*green*) or 300 s (*red*) of I and T. Small inward tail currents at the time indicated by the *dashed box* are shown at a higher magnification in the *inset*. The *bottom panel* shows the voltage protocol. *Vertical* and *horizontal scales* are the same in all current traces (except *inset*). *Dashed horizontal black lines* indicate the zero current level. *B,* current amplitudes (end-pulse minus beginning of pulse current) plotted against time. Each *symbol* represents the amplitude of current during a single pulse. The time at which I and T and astemizole were applied is indicated by *vertical arrows*. Current traces and current amplitudes in *Ai, Aii,* and *B* were measured in the same oocyte. *C,* fluorescence imaging of Ca^2+^*_i_* by confocal microscopy. Fluorescence images from a representative oocyte loaded with the Ca^2+^-sensitive indicator Oregon Green 488 BAPTA-1 were taken before (control) and at indicated times after I and T application. *Scale bar* on *left panel* is 200 μm. *D,* time course of changes of fluorescence following I and T application. The fluorescence signal (*F*) was normalized to levels prior to I and T application (*F*_0_) and plotted against time after compound addition. The time scale is the same as for *B. Symbols* represent mean levels (*n* = 9) at 5-s intervals, and *dotted lines* indicate ± S.E.

To characterize the response of wild-type hEAG1 currents to elevated Ca^2+^*_i_*, oocytes were voltage-clamped, and 2-s voltage steps to +60 mV were applied repetitively at 10-s intervals from a holding potential of −90 mV. hEAG1 currents were characterized by relatively slow activation. To elevate Ca^2+^*_i_*, the Ca^2+^ ionophore, ionomycin (I), and the sarco/endoplasmic reticulum ATPase inhibitor, thapsigargin (T), were applied. hEAG1 currents were initially profoundly inhibited by bath application of 5 μm I and T (Fig. 2*Ai*). Mean maximal inhibition was 74.3 ± 2.3%, and mean time to maximal inhibition was 86.7 ± 7.2 s (*n* = 21). After the initial inhibition, the hEAG1 current slowly started to increase in amplitude to a mean level after 300 s of 43 ± 4% of the control current amplitude, despite the continued presence of I and T (Fig. 2 and Table 1). Fluorescence imaging of Ca^2+^*_i_* in *Xenopus* oocytes by confocal microscopy revealed that Ca^2+^*_i_* was rapidly elevated by I and T. A ring-shaped fluorescence pattern was observed as a result of the light-absorbing properties of the oocyte yolk sac and thus the loss of signal from the deeper central portion of the cell (Fig. 2*C*). Importantly, cytoplasmic Ca^2+^ was sustained at a high level, which we estimate to be ∼1 μm (see “Experimental Procedures”). In most cells, Ca^2+^-dependent fluorescence reached a plateau and did not decline in the sustained presence of I and T. These results strongly suggest that the slow recovery of the hEAG1 current is not due to Ca^2+^*_i_* returning to resting levels.

**TABLE 1:** **Response of WT and mutant hEAG1 currents to I and T** The 2nd column gives the mean values for maximum responses (either inhibition or potentiation) calculated as current amplitudes in I and T divided by control currents. The 3rd column gives the mean values for responses at 300 s, to quantify changes of current amplitudes during the course of experiments where they have occurred. If the response is maximal after 300 s this is indicated by the abbreviation, Max, and the value given in the maximum response column.

Construct	Fold change of amplitude with I and T		*n*
	Maximum Response	Response after 300-s	
WT hEAG1	0.15 ± 0.02	0.43 ± 0.04	21
F714S/F717S	0.76 ± 0.09	1.01 ± 0.03	7
F151N/L154N	0.39 ± 0.04	Max	7
ΔeagD	1.82 ± 0.37	Max	6
ΔcNBHD	1.07 ± 0.03	Max	7
ΔPAS-cap	15.7 ± 1.42	2.84 ± 0.69	9
ΔPAS-cap/F714S/F717S	1.03 ± 0.04	Max	13
WT (EGTA)	1.01 ± 0.01	1.02 ± 0.02	5
E600R	12.25 ± 2.61	Max	5
E600A	0.24 ± 0.04	0.38 ± 0.08	7
E600Q	0.26 ± 0.03	0.41 ± 0.05	14
E600L	3.16 ± 0.60	Max	13
E600I	6.43 ± 0.72	Max	11

The current observed with sustained application of I and T was characterized by very slow activation kinetics. Mean *t*_10–80%_ at +60 mV was 1201 ± 93 ms, which was significantly longer than control values of 422 ± 25 ms (*p* < 0.0001, *n* = 8). Like the control current, it was profoundly blocked by 50 μm astemizole, a well characterized hEAG channel blocker. *Xenopus* oocytes are known to express Ca^2+^*_i_*-activated Cl^−^ currents, but these are unlikely to significantly contribute to the outward currents at +60 mV in our low Cl^−^ recording solutions as the driving force for Cl^−^ flux was small (∼10 mV). Outward Ca^2+^*_i_*-activated currents were also negligible in un-injected or water-injected oocytes (Fig. 2*Aiii*). Furthermore, although I- and T-dependent inward tail currents were observed in many cells at the −90 mV holding potential (when Cl^−^ driving force is ∼160 mV), these rapidly declined, whereas the hEAG1 current increased. We excluded data from cells with inward tail currents greater than 1 μA to further minimize the potential for contamination of hEAG1 currents.

**FIGURE 3.**
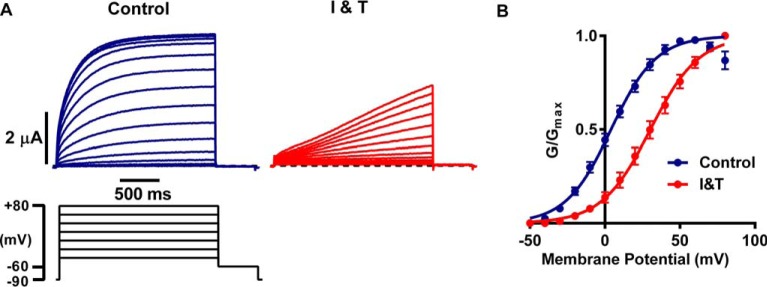
**With sustained high Ca^2+^*_i_* hEAG1 current inhibition is reduced but activation gating is slowed and shifted to depolarized potentials.** *A,* representative WT hEAG1 current traces before (*blue*) and >300 s after I and T application (*red*) elicited by an I-V protocol consisting of 2-s pulses from −50 to +80 mV at 10-mV increments from a holding potential of −90 mV, with a 500-ms step to −60 mV after each test potential. The voltage protocol is illustrated in the *bottom left-hand panel* (not all voltage steps are shown). *B,* mean (± S.E., *n* = 6) conductance-voltage relationships for WT hEAG1 currents before (*blue symbols*) and during I and T application (*red symbols*), fitted with Boltzmann functions (*solid lines*). Conductance is normalized to maximum for control and I and T (*I&T*) values.

Representative traces of hEAG1 currents measured in response to an I-V protocol in control solution and after >300 s in I and T are shown in Fig. 3*A*. In control solution, hEAG1 currents were activated at potentials positive to −40 mV. Conductance reached a peak at +60 mV and exhibited a small decrease at more positive potentials, consistent with a small amount of inactivation that has been described by others (32, 33). In comparison, the activation threshold for currents in I and T was shifted to depolarized potentials (positive to −20 mV), and the currents were far slower to activate and showed no rectification. The mean relationships for voltage dependence of isochronal activation in control and I and T solutions are shown in Fig. 3*B*. Activation was shifted by +29 mV (*p* < 0.0001, *n* = 6, see Table 2). Qualitatively similar responses were observed with 2 μm lysophosphatidic acid (data not shown), which stimulates G_q_-coupled receptors and thus increases cytosolic calcium through inositol trisphosphate-dependent release from the endoplasmic reticulum and subsequent store-operated Ca^2+^ entry. Taken together, these results reveal a multiphasic response of WT hEAG1 currents to I and T, consisting of an initial rapid inhibition, followed by a second delayed phase during which current amplitudes start to increase, but activation is slowed and shifted to depolarized potentials by a Ca^2+^*_i_*-dependent stabilization of the closed state.

**TABLE 2:** **Voltage- and time-dependent kinetics of WT and mutant hEAG1 channel currents** Values (mean ± S.E.) for voltage at which channels are activated by 50% (*V*_0.5_) and slope (*k*) of the relationship are given, along with times from 10 to 80% activation (*t*_10–80%_). ND indicates not determined.

Construct	Control		I and T		*n*	Control, *t*_10–80%_	I and T, *t*_10–80%_	*n*
	*V*_0.5_	*k*	*V*_0.5_	*k*				
WT hEAG1	4.1 ± 2.1	14.8 ± 0.9	33.0 ± 3.7	19.1 ± 2.1	6	422 ± 25	1201 ± 93	8
F714S/F717S	−0.3 ± 3.0	18.7 ± 2.6	12.8 ± 2.8	19.29 ± 2.4	8	387 ± 16	681 ± 82	6
F151N/L154N	51.0 ± 1.4	21.2 ± 1.0	ND	ND	8	316 ± 119	ND	5
Δ2-135	−4.3 ± 0.6	11.6 ± 0.7	−5.1 ± 3.1	15.5 ± 3.7	5	952 ± 67	962 ± 46	5
ΔcNBHD	35.6 ± 1.6	23.0 ± 1.0	40.2 ± 1.4	23.4 ± 1.4	7	459 ± 29	453 ± 24	7
ΔPAS-cap	−11.5 ± 2.6	12.6 ± 0.9	−12.5 ± 4.0	18.4 ± 1.7	7	656 ± 31	951 ± 58	9
ΔPAS-cap/F714S/F717S	−14.5 ± 2.8	12.7 ± 1.1	−18.2 ± 3.0	14.4 ± 1.1	10	551 ± 19	678 ± 47	9
WT (EGTA)	6.9 ± 1.6	26.0 ± 2.7	16.4 ± 3.4	25.0 ± 1.5	5	ND	ND	

##### hEAG1 Inhibition by Ca^2+^_i_ Is CaM-mediated

**FIGURE 4.**
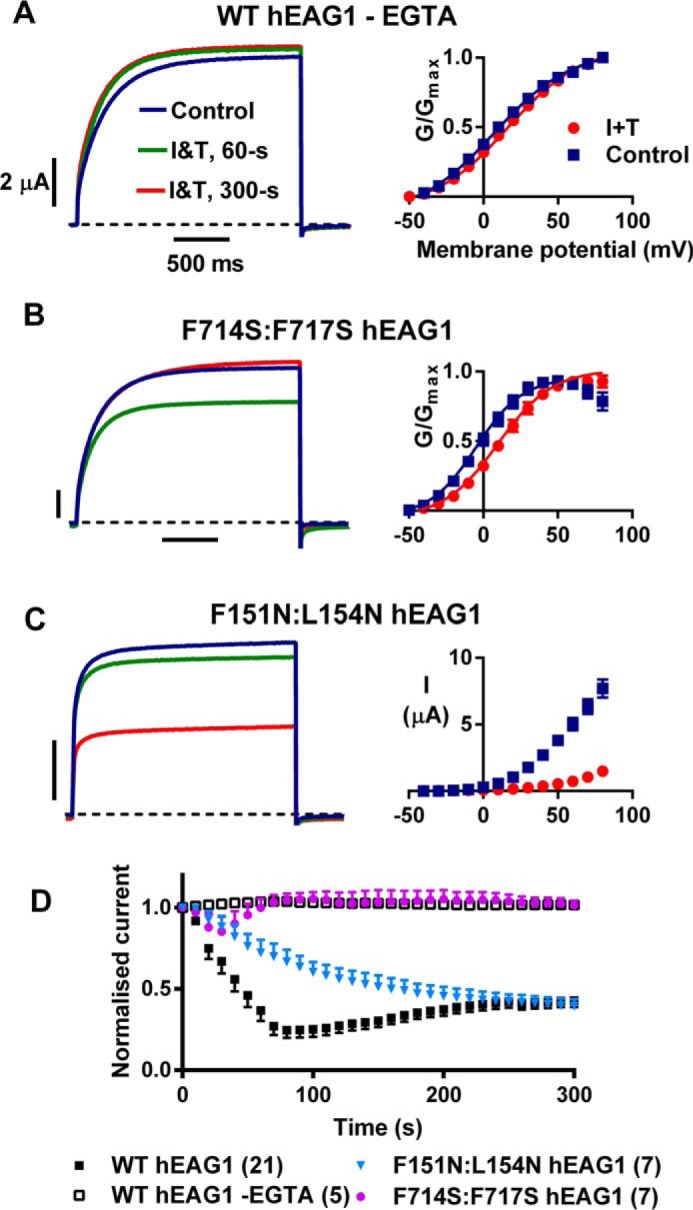
**Response of hEAG1 currents to Ca^2+^*_i_* is calmodulin dependent.** *A–C, left-hand panels,* representative current traces with voltage pulses to +60 mV before and at indicated times following I and T (*I&T*) application. *A–C, right-hand panels,* mean (± S.E.) conductance-voltage relationships fitted with Boltzmann functions (*A* and *B*) or current amplitude relationships (*C*). *Error bars* that are too small to extend beyond the symbols are not shown. *A,* responses in oocytes expressing WT hEAG1 channels and injected with EGTA (estimated final concentration 5 mm, *n* = 5). *B,* responses from oocytes expressing F714S/F717S hEAG1, a mutant that reduces the affinity for Ca^2+^-CaM binding to the BD-C2 domain (*n* = 7). *C,* responses in F151N/L154N hEAG1, a mutant with reduced affinity for Ca^2+^-CaM binding to the BD-N1 domain (*n* = 7). *D,* time courses of changes in WT and mutant hEAG1 currents after switching to I and T containing the bath solution. Time-dependent currents with each voltage step to +60 mV were normalized to control current and mean (± S.E.) values plotted against time from switching to I and T. Numbers (*n*) are indicated in *parentheses* next to symbol legends.

Additional experiments were performed to confirm that the observed responses to I and T were Ca^2+^-CaM mediated. First, we examined the effect of buffering Ca^2+^*_i_* with 5 mm EGTA. Currents were first recorded under control conditions and then, while the oocyte was still voltage-clamped, EGTA was injected into the cell via a micropipette, and the response to I and T was recorded. With EGTA present, the current amplitudes were slightly increased rather than being inhibited by I and T, and there were no significant changes in the *V*_0.5_ and slope values for the voltage dependence of activation (*p* > 0.05, *n* = 5). To investigate whether the inhibition of WT hEAG1 was dependent on CaM binding to the channels, we tested the effect of I and T on mutants that reduce the binding affinity of CaM to either BD-N or BD-C2 (13, 31), the high affinity CaM-binding sites. The BD-C1 site binds CaM in the micromolar range and was not included. The F714S/F717S BD-C2 mutant displayed similar gating to WT hEAG1 under control conditions but showed considerably attenuated responses to I and T (Fig. 4*B*). There was an initial small inhibition (Fig. 4*D* and Table 1), but the current then quickly rebounded to control amplitudes, and the effect on *t*_10–80%_ activation at +60 mV (271 ± 74, *n* = 6) was significantly reduced (*p* < 0.005) compared with WT hEAG (820 ± 77, *n* = 8). The shift in isochronal activation was +13 mV, which was significantly smaller than for WT hEAG1 (*p* < 0.005). Interestingly, the F151N/L154N BD-N mutations had quite a different effect compared with BD-C2 mutations. The voltage dependence of activation was +47 mV more positive than WT hEAG1 in control solution, suggesting that CaM binding at this site influences channel function at basal/resting Ca^2+^*_i_* levels. The *t*_10–80%_ activation at +60 mV was also faster than WT hEAG (see Table 2). The time course of the response to I and T was also profoundly different. There was no initial fast component of inhibition as observed in WT hEAG1, and instead inhibition developed slowly and progressively (Fig. 4*D*), without there being any marked slowing of time-dependent activation kinetics. The voltage dependence of activation was shifted too positive to quantify *V*_0.5_ during I and T, but the threshold for activation was 50 mV more depolarized than under control conditions (Fig. 4*C*). Collectively, these results indicate that I and T exert their effects through a Ca^2+^-CaM-mediated process and that CaM is probably influencing channel gating under resting Ca^2+^*_i_* conditions. They also indicate that there is a complex interplay between the CaM binding domains, with BD-N and BD-C2 each regulating different aspects of channel inhibition in response to Ca^2+^-CaM.

##### The Eag Domain and cNBHD Are Required for Ca^2+^-CaM Regulation of hEAG1

**FIGURE 5.**
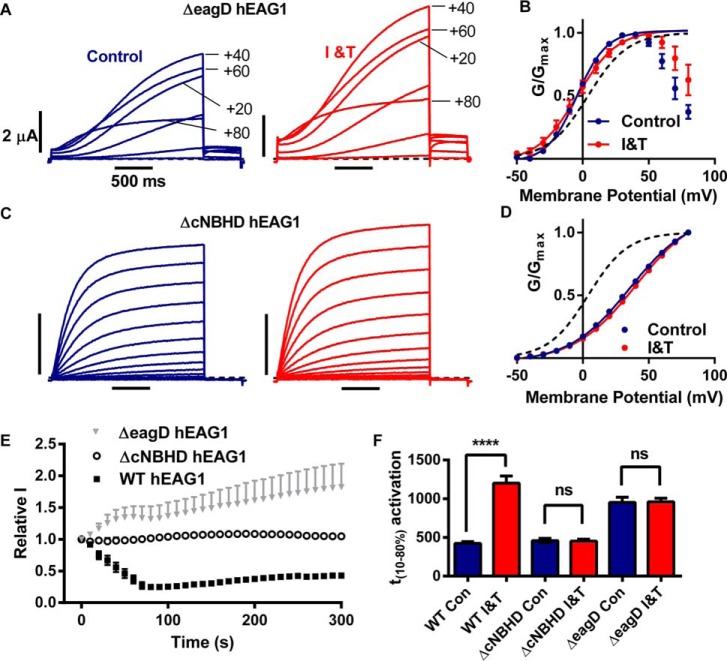
**eagD and cNBHD are both required for Ca^2+^-CaM-dependent hEAG1 current inhibition.** *A* and *C,* representative traces of ΔeagD hEAG1 (*A*) and ΔcNBHD hEAG1 (*C*) currents elicited by *I-V* protocols before (*left panels*) and during I and T (*I&T*) (*right panels*) application for >300 s. ΔeagD hEAG1currents exhibited rectification. For clarity, only selected traces, elicited by voltage steps +20 mV apart, are shown. *B* and *D,* mean (± S.E.) conductance-voltage relationships for ΔeagD hEAG1 (*B*, *n* = 5) and ΔcNBHD hEAG1 (*D*, *n* = 7) currents before (*blue symbols*) and during I and T application (*red symbols*), fitted with Boltzmann functions (*solid lines*). The voltage dependence of activation for WT hEAG1 in control solution is shown for comparison (*black dashed line*). *E,* mean (± S.E.) normalized current amplitudes (see Fig. 3*D* for details) for Δ2–135 hEAG1 (*n* = 6) and ΔcNBHD hEAG1 (*n* = 7) plotted against time after switching to I and T. The time course of WT hEAG1 (*n* = 21) is shown for comparison. *F,* time between 10 and 80% activation (*t*_10–80%_) at +60 mV in the presence (*red*) or absence (*blue*) of elevated Ca^2+^*_i_* values for WT (*n* = 8), ΔcNBHD hEAG1 (*n* = 7), and ΔeagD hEAG1 (*n* = 5). ****, *p* < 0.0001. *ns, p* > 0.05.

To test whether the hEAG1 channel response to Ca^2+^-CaM was mediated by interactions between the eag domain and cNBHD, we next tested the effect of deleting each structural domain in turn on Ca^2+^-CaM-dependent gating. Deleting the eagD (amino acids 2–135) dramatically altered gating and resulted in a slowly activating and slowly deactivating current. At potentials positive to +40 mV, current amplitudes progressively decreased and the tail currents, which also had a smaller peak amplitude, had a 'hooked' appearance (Fig. 5*A*). This behavior resembles hERG channel gating, in which the rectification is due to rapid onset of inactivation, and the hooked tails are due to rapid recovery from inactivation followed by slow deactivation. Importantly, in ΔeagD hEAG1, the inhibition by elevated Ca^2+^*_i_* was completely abolished; instead, the current was significantly increased by 82 ± 37% (*p* < 0.005, *n* = 7, see *I-V* relationship in Fig. 5*A* and mean time course data in Fig. 5*E*). The effect of I and T on the voltage and time dependence of ΔeagD activation was also significantly attenuated compared with WT hEAG1 (*p* < 0.0001, Fig. 5, *B* and *F*).

Deleting the cNBHD and C-linker (amino acids 484–668) also abolished all effects of elevating Ca^2+^*_i_* on hEAG1 current gating (Fig. 5, *C–F*). Activation gating was also shifted by +32 mV compared with WT hEAG1. These results indicate that the eag domain and cNBHD have important roles in both voltage and Ca^2+^*_i_*-CaM-dependent gating.

##### hEAG1 Channels Lacking the PAS-cap Are Potentiated Rather than Inhibited by Ca^2+^_i_

**FIGURE 6.**
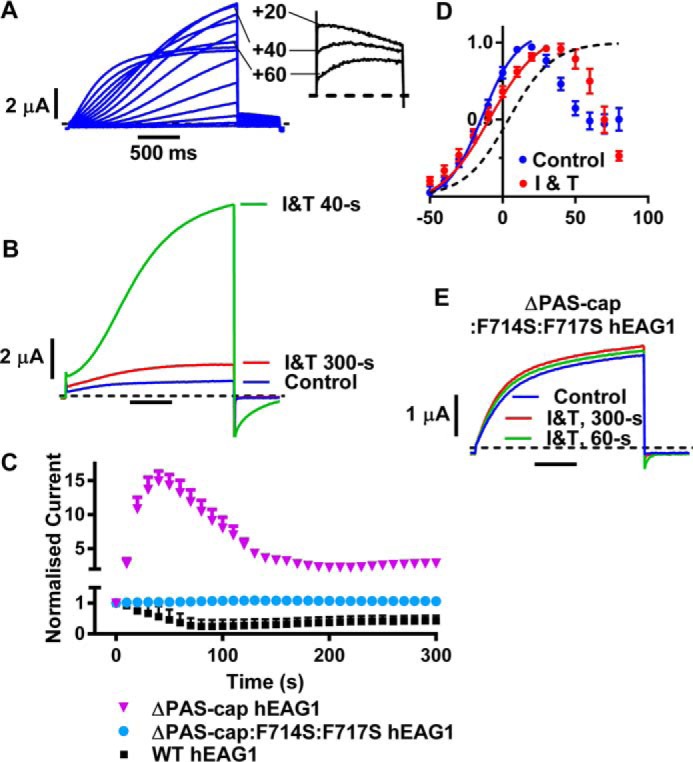
**PAS-cap is a critical regulatory domain for both voltage- and calcium-dependent hEAG1 channel gating.** *A,* representative ΔPAS-cap hEAG1 current traces elicited by test potentials between −50 and +80 mV. Note the progressive reduction of current amplitudes at potentials positive to +40 mV. *Inset* shows tail currents recorded at −60 mV following test potentials to +60, +40, and +20 mV. *B,* representative ΔPAS-cap hEAG1 current traces in control solution (*blue trace*) and 40 s (*green trace*) or 300 s (*red trace*) after applying I and T (*I&T*). Currents were elicited with voltage steps to +60 mV from a holding potential of −90 mV. I and T caused an initial profound potentiation of ΔPAS-cap hEAG1 current that was in stark contrast to the inhibition of WT hEAG1. The inward tail current observed in the trace after 40 s of I and T is likely to be due to extracellular K^+^ accumulation following large amplitude test pulse currents. The currents in *A* and *B* are from different cells. *C,* mean (± S.E.) normalized current amplitudes (see Fig. 3*D* for details) for ΔPAS-cap hEAG1 (*n* = 9) and ΔPAS-cap/F714S/F717S hEAG1 (*n* = 14) plotted against time after switching to I and T. The time course of WT hEAG1 is also shown for comparison (*n* = 21). *D,* mean (± S.E.) conductance-voltage relationships for ΔPAS-cap hEAG1 (*n* = 7) before (*blue symbols*) and during I and T application (*red symbols*), fitted with Boltzmann functions (*solid lines*). *Black dotted line* shows the activation curve for WT hEAG1 for comparison with mutants. *E,* representative ΔPAS-cap/F714S/F717S hEAG1 currents with voltage pulses to +60 mV before and at indicated times after I and T application.

The eagD can structurally be divided into the PAS domain (amino acids 27–135) and the PAS-cap (residues 1–26). The PAS-cap is highly conserved across the KCNH channel family (Fig. 1*B*), and functional studies have demonstrated that much of the regulation of both hEAG1 and hERG1 voltage-dependent gating attributed to the eagD is actually mediated by the PAS-cap. Consistent with this, deleting the PAS-cap results in currents (see Fig. 6*A*) with gating properties almost identical to when the entire eagD is deleted. ΔPAS-cap and ΔeagD hEAG1 currents both exhibit a profound slowing of activation and deactivation and increased rectification at positive potentials. To determine whether the PAS-cap is also functionally important for Ca^2+^-CaM-dependent gating, we investigated the response of ΔPAS-cap hEAG1 currents to I and T. Currents were elicited by repetitively stepping to +40 mV, and the responses before and during I and T are shown in Fig. 6*B*. Surprisingly, ΔPAS-cap hEAG1 currents were substantially *increased* by I and T application, with an average peak change of current of 15.70 ± 1.42-fold relative to the control currents (*n* = 9). This is in stark contrast to the 75% *inhibition* of WT hEAG1. The potentiation of current in the continued presence of I and T reached a peak after a mean time of 43.3 ± 4.7 s (*n* = 9) and then started to decline, although amplitudes stabilized at a level that was still 2.84 ± 0.69-fold larger (*n* = 9) than the control current (Fig. 6*C*). The time courses of the ΔPAS-cap and WT hEAG1 responses to I and T are almost a mirror image of one another. ΔPAS-cap is potentiated, whereas WT hEAG1 is inhibited, but they both show an initial fast change, which is transient in nature, followed by a relatively slow “recovery” phase. This suggests that both channels are Ca^2+^*_i_*-sensitive and that there may be common underlying mechanisms. The results also demonstrate that the PAS-cap region is absolutely required for “latching” the channel in a non-conducting state. To confirm that the underlying mechanism of PAS-cap hEAG1 current potentiation was mediated by CaM binding, we simultaneously mutated the CaM BD-C2 site. Representative currents for ΔPAS-cap/F714S/F717S hEAG1 before and during I and T are shown in Fig. 6*E* and the mean time courses in Fig. 6*C*. This mutant was insensitive to I and T with no significant change of *V*_0.5_ and *t*_10–80%_ of activation or current amplitude compared with control (*p* > 0.05, *n* = 7–9), thus confirming the Ca^2+^-CaM dependence of ΔPAS-cap hEAG1.

##### Ca^2+^-CaM-dependent Inhibition of hEAG1 Required Interactions between the PAS-cap and cNBHD

**FIGURE 7.**
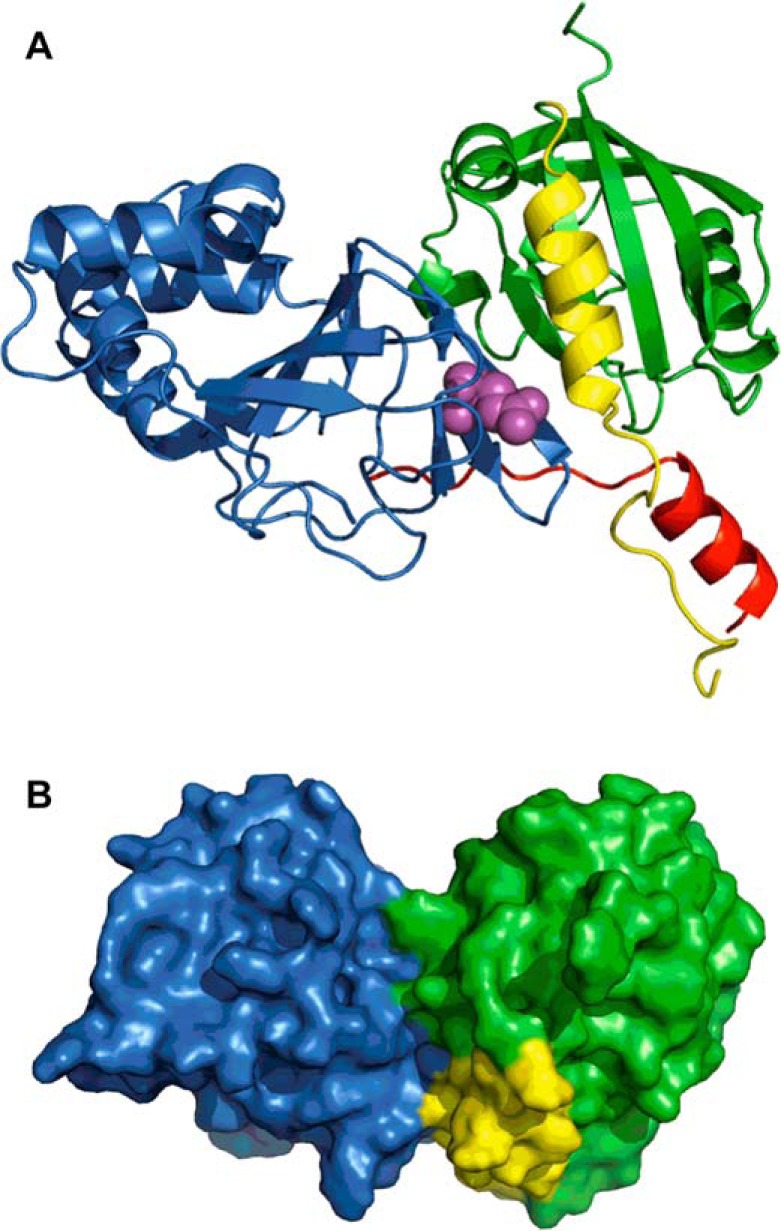
**Molecular model of interactions between cNBHD and PAS-cap of hEAG1.** *A,* homology model of the eagD-cNBHD complex based upon the crystal structure (Protein Data Bank code 4LLO (25)). The PAS-cap is shown in *yellow*, the PAS domain in *green*, the cNBHD in *blue,* and the post-cNBHD region in *red*. The side chain of Glu-600 is shown in *spheres* and colored *magenta. B,* homology model rotated around the horizontal axis by ∼90° with surface rendering to illustrate how the amphipathic α-helix of the PAS-cap sits between the PAS domain and cNBHD. Colors correspond to *A*.

**FIGURE 8.**
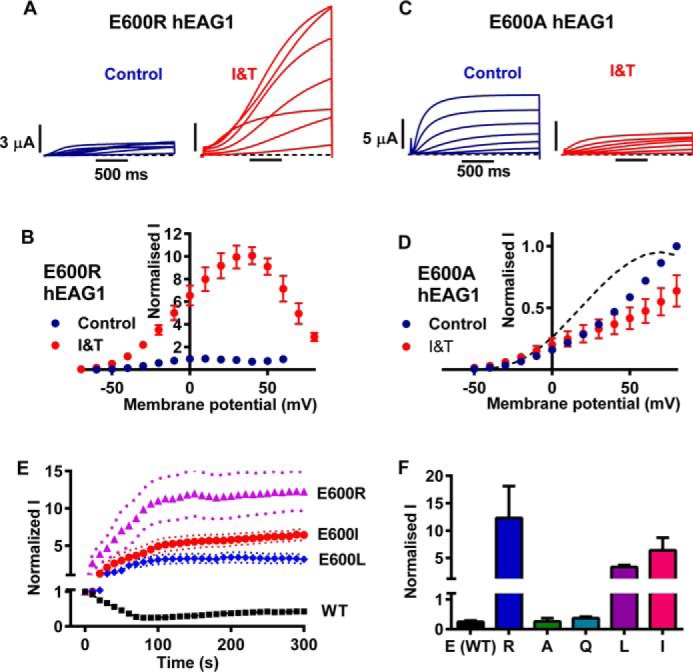
**Point mutations to the cNBHD mimic the effect of deleting the PAS-cap on voltage- and calcium-dependent properties.** *A* and *C,* representative current traces elicited by the *I-V* protocol for E600R (*A*) and E600A (*C*) hEAG1 before (control, *blue traces*) and during I and T (*I&T*) application (*red traces*). Traces at +20-mV increments are shown for clarity. *B* and *D,* mean (± S.E.) normalized current-voltage relationships for E600R (*B*, *n* = 7) and E600A (*D*, *n* = 8) hEAG1. Time-dependent currents at each potential were normalized to the maximum current in control conditions to illustrate the fold-change of current amplitude in response to I and T. The *dotted lines* in *D* show the mean normalized relationships for WT hEAG1 currents for comparison. *E,* mean (± S.E.) normalized to control current amplitudes against time in I and T for cNBHD point mutants E600R (*n* = 5), E600A (*n* = 7), E600L (*n* = 13), E600I (*n* = 11), and WT hEAG1 (*n* = 21). Note the split current axis to accommodate the substantial potentiation of currents exhibited by E600R, E600I, and E600L hEAG1. *Dotted lines* indicate ± S.E. *F,* mean maximum changes of current for Glu-600 mutants and WT hEAG1 in response to I and T, normalized to control current amplitudes. *n* = 7 and 14 for E600A and E600Q, respectively, and the other numbers (*n*) are the same as in *E*. The normalized current axis has been split for the same reason as given in *E*.

Recently, an x-ray crystal structure of the eag domain in complex with the cNBHD of mEAG has been solved (25). There are a number of sites of contact between the two domains, including contact of the PAS domain with the intrinsic ligand and post-cNBHD motifs of the cNBHD, which are adjacent to the BD-C1 and BD-C2 CaM-binding sites of the channel. The other important region of contact is between the PAS-cap and the cNBHD, although it should be noted that the first 16 amino acids of the PAS-cap were not resolved. Interestingly, there are relatively few structural changes between the domains in the complex and those of the isolated domains when they were solved independently of one another. This suggests that the contacts are quite weak and consistent with the low binding affinity (13.2 μm) measured using fluorescence anisotropy (25). Other regions of the channel may both stabilize and regulate the interaction of these crucial regions. Of particular interest was that a number of disease-associated mutations (long QT causing mutations in hERG1 and cancer-linked mutations in hEAG1) mapped to the interface between the eag domain and cNBHD. We modeled the interaction of the hEAG1 eag domain in complex with the cNBHD, including the section of the PAS-cap missing in the crystal structure (Fig. 7). The PAS-cap folds back, and the amphipathic helix sits in a groove between the PAS domain and cNBHD, making contacts with both domains. To test whether the Ca^2+^-CaM-mediated inhibition of hEAG1 could be due to interactions of the PAS-cap with the cNBHD, we mutated Glu-600 (equivalent to Glu-627 in mEAG and Glu-788 in hERG1) to Ala, Arg, Gln, Ile, or Leu and tested the response of these point mutants to I and T. This position is highly conserved in the EAG channel family and mutations in the homologous hERG1 channel position are associated with long QT syndrome (34). In mEAG, Ala and Arg mutations have been reported to cause robust depolarizing shifts in the voltage dependence of activation of >100 mV (25). In hEAG1, the effects were not as marked but, remarkably, the E600R mutation reproduced much of the change in gating properties (slowed activation and deactivation, and rectification at positive potentials) seen in ΔPAS-cap hEAG1 (Fig. 8, *A* and *B*). The substantial Ca^2+^*_i_*-dependent potentiation was also seen with the cNBHD E600R mutant. I and T resulted in a 12.3 ± 2.6-fold increase in E600R hEAG1 current measured at +40 mV relative to control (Table 1). However, unlike the ΔPAS-cap mutant, the increase of current in I and T was sustained (Fig. 8*E*). The response of Glu-600 mutants to elevated Ca^2+^*_i_* was highly dependent on residue volume and not just the charge of the substituted residue. The E600A and E600Q mutants exhibited WT hEAG1 behavior and were inhibited by I and T (Fig. 8*F* and Table 1). Ala and Gln have van der Waals residue volumes of 67 and 96 Å^3^, respectively, which are smaller than Glu (109 Å^3^) (35). Substituting Glu-600 for Leu or Ile (each 124 Å^3^) resulted in potentiation of current by I and T of 3.16 ± 0.6 (*n* = 13) and 6.43 ± 0.72 (*n* = 11)-fold, respectively. The volume of Arg is largest of all (196 Å^3^) and gave the greatest potentiation. Collectively, these results suggest that Glu-600 is an important site of contact with the PAS-cap. They also suggest that the charge of the residue is not the critical factor. The functional data support a molecular model in which the PAS-cap packs against the cNBHD. Substitution of large residues at position 600 reduces PAS-cap binding affinity resulting in similar functional effects to deleting the PAS-cap entirely.

In recent years, studies have shown the importance of interactions between the eagD and cNBHD for regulating voltage-dependent gating of both hEAG1 and hERG1 channels (17, 25, 28–30). The cNBHD is directly coupled to the S6 inner helix of the pore by the C-linker. A similar structural motif is also found in CNG and HCN, and in these channels it is crucial for their cyclic nucleotide-dependent regulation (36–38). It seems likely that conformational changes in the cNBHD of hEAG1 and hERG1 channels will also be transduced to the pore via the C-linker. Interactions of the eag domain with the cNBHD regulate the gating of both hEAG1 and hERG1 channels but in a very different manner. In hERG1, the interaction between the two domains slows deactivation gating by stabilizing the open conformation of the channel (17, 28). The eagD also influences hERG1 inactivation, such that onset of inactivation is faster, and recovery from inactivation is slower when the eagD is present compared with when it has been deleted (22, 39, 40). For hEAG1 channels, the eagD has the opposite effects on gating. Because the PAS-caps are almost identical, it seems likely that the differential roles of the eagD and cNBHD are mediated by differences in how the PAS-cap binds to the PAS domain and cNBHD. The interactions are dynamic and influence both voltage-dependent gating and, in the case of hEAG1, Ca^2+^-CaM-dependent gating. hERG1 channels are not regulated by Ca^2+^-CaM-mediated mechanisms (data not shown).

This study provides mechanistic insight into how hEAG1 channels are regulated by Ca^2+^*_i_*. The eagD and cNBHD are both required for transducing the effect of Ca^2+^-CaM binding. Further studies are required to elucidate the precise molecular mechanisms. Our results suggest a complex sequence of events, with distinctive roles for the BD-C2 and BD-N CaM binding domains and the PAS-cap, PAS, and cNBHD structural domains. In the WT hEAG1 channel, raising Ca^2+^*_i_* results in a multiphasic response consisting of an initial inhibition followed by a slow “recovery” phase in which not only are current amplitudes returning to control levels but activation gating is profoundly slowed and shifted to depolarized potentials. It seems likely that different components of the transduction mechanism coupling CaM binding to changes of gating of the pore are involved in regulating this process. The PAS-cap is an integral component. When deleted, the hEAG1 channels remain Ca^2+^*_i_*-sensitive, but instead of being inhibited, they are potentiated by more than 15-fold. Hypothetically, the effects of deleting the PAS-cap could be due to alterations of PAS domain structure, leading to allosteric effects on PAS domain interactions with the cNBHD or other structural domains. However, mutations of Glu-600 on the cNBHD cause a similar substantial potentiation, suggesting the PAS-cap interacts directly with this critical site and that this PAS-cap interaction with the cNBHD is required for stabilizing the closed state of the channel. Without this interaction, channel activity is very high. Nevertheless, there is one clear difference. Deletion of the PAS-cap causes a peak and plateau type of response, whereas mutations of the cNBHD cause a sustained potentiation. Thus, Glu-600 cNBHD mutations also destabilize conformations that occur later on when elevated Ca^2+^*_i_* levels are sustained. Mutations to the CaM-binding sites also have differential effects. Mutating BD-N results in only a slow onset of inhibition, perhaps because CaM binding at this site is required for early conformational changes that lead to the fast inhibition. In contrast, when the BD-C2 site is mutated, the currents still show an initial early response to elevated Ca^2+^*_i_*, but then they fully recover to control amplitudes, suggesting that this site is required for stabilizing conformations that occur with elevated Ca^2+^*_i_*.

hEAG1 is one of a number of ion channels that are modulated by Ca^2+^*_i_* in a CaM-dependent manner (41, 42). Several of these channels are also modulated by phosphoinositide 4,5-bisphosphate (PIP_2_) (43). There is growing evidence that in TRPC6, and other channels, phosphoinositides can bind at, or close to, the CaM-binding site and regulate CaM binding, thus providing a mechanism to integrate these two important second messenger pathways (44). In type 2 small conductance Ca^2+^*_i_*-activated potassium (SK2) channels, PIP_2_ is a cofactor for Ca-CaM activation of these channels and binds at the interface between CaM and SK2 at a site that includes residues from both proteins (45, 46). The phosphorylation status of CaM itself may also be dynamically regulated by a signaling complex on the SK2 channel (44). Phosphorylation of CaM results in a reduced affinity of PIP_2_ for the CaM-SK2 complex, causing channel inhibition (46). Recently, hEAG1 channels have also been shown to be regulated by PIP_2_ (47). In this case, PIP_2_ inhibits hEAG1 channels through a mechanism that requires an intact CaM BD-N region (47). Whether there is integration of CaM and PIP_2_ signaling in hEAG1 channels via overlapping interactions with BD-N remains to be determined.

##### What is the Physiological Importance of Ca^2+^-CaM-dependent Regulation of hEAG1?

hEAG1 channels are expressed in presynaptic terminals of the CNS (4, 48, 49). mEAG1 knock-out mice exhibit increased levels of presynaptic Ca^2+^*_i_* in response to sustained trains of high frequency APs compared with WT mice. This in turn leads to enhanced neurotransmitter release and faster rates of increase and larger amplitudes of excitatory post-sypaptic currents (4). This, together with other findings, suggests that EAG channels become activated during bursts of presynaptic APs, shortening local APs at synaptic terminals and modulating synaptic plasticity. It seems reasonable to suggest that this function of EAG1 channels will be regulated not only in a firing frequency, but also in a Ca^2+^*_i_*-dependent manner. Expression of hEAG1 is also dramatically increased in several types of cancer. Indeed, mutations at the interface of the eag domain-cNBHD complex have been linked to this disease (25). A recent report suggests that the role of hEAG1 in tumorigenesis is the disassembly of the primary cilium, a microtubule-based structure and specialized calcium compartment that needs to be taken apart prior to mitosis (50). Further studies are needed to determine what role the exquisite Ca^2+^*_i_* sensitivity of these channels has in normal health as well as diseases such as cancer. However, understanding the molecular interactions that regulate hEAG1 channels could help in the design of novel therapies that mimic Ca^2+^-CaM inhibition of hEAG1 to selectively target hEAG1-expressing cancer cells.

## Experimental Procedures

### 

#### 

##### Site-directed Mutagenesis and Electrophysiology

Site-directed mutagenesis was performed using the QuikChange mutagenesis technique (Stratagene, La Jolla, CA) on hEAG1 subcloned into pXOOM (17), a kind gift from Dr. Thomas Jespersen, University of Copenhagen (51). Plasmid DNA was linearized with XbaI, and *in vitro* transcription was performed using T7 RNA polymerase (mMessage mMachine, Ambion, Austin, TX). *Xenopus laevis* oocytes were isolated, defolliculated, maintained in culture, and injected with wild-type or mutant cRNA (0.05 to 3 ng per oocyte) as described previously (52). Whole cell currents were recorded in *Xenopus* oocytes using a two-electrode voltage clamp (52, 53). Microelectrodes were filled with 3 m KCl, and the tips were broken to give resistances of 1.1–1.5 megohms. Recordings were made at room temperature 1–5 days after cRNA injection. Data were low pass filtered and sampled at 5 kHz and saved to a computer for off-line analysis using a Digidata 1320A data acquisition system (Molecular Devices, Sunnyvale, CA). Oocytes were perfused with a low chloride, MES-based solution containing (in mm) Na-MES 96, K-MES 2, Ca-MES_2_ 2, MgCl_2_ 1, HEPES 5, pH 7.6. The low chloride solution minimized endogenous outward calcium-activated chloride currents. We estimate *E*_Cl_ to be ∼+70 mV, based on extracellular Cl^−^ of 2 mm, and reported intracellular concentrations of 40–45 mm (54). Unless stated otherwise, cytoplasmic calcium was elevated by perfusing cells with supermaximal concentrations (5 μm) of ionomycin and thapsigargin purchased from Sigma (United Kingdom) or Santa Cruz Biotechnology (Dallas, TX). In some experiments, EGTA was injected into the oocytes during recordings to buffer Ca^2+^*_i_* to low levels. 50 nl of 50 mm K_4_EGTA solution, pH 7.2, was injected using a Nanoliter 2000 microinjection device (World Precision Instruments, Sarasota, FL) to give an estimated final concentration of 5 mm based on the assumption that oocytes have a cytoplasmic volume of 500 nl.

##### Voltage Protocols and Data Analysis

The time- and voltage-dependent kinetics of hEAG1 were measured using an *I-V* protocol, which unless stated otherwise consisted of 2-s test pulses to potentials between −50 and +80 mV followed by a 500-ms pulse to −60 mV. Responses to elevated Ca^2+^*_i_* were monitored by repetitively stepping to +60 or +40 mV for 2 s. In all experiments the holding potential was −90 mV, and pulses were applied at 10-s intervals.

hEAG1 current amplitudes were measured using Clampfit software (Molecular Devices, Sunnyvale, CA). Conductance (*G*) was calculated as the time-dependent current amplitude (or end-pulse current amplitude for Δeag and ΔPAS-cap) in response to a test pulse (*V_m_*), divided by driving force (*V_m_* − *E*_K_), where *E*_K_ is the equilibrium potential for K^+^ (−96 mV). For each oocyte, *G* was normalized to *G*_max_ (maximum conductance) plotted as a function of *V_m_* and fitted to a Boltzmann function to determine the potential at which the current is half-maximally activated (*V*_0.5_) and the slope (*k*) of the conductance-voltage relationship. For mutants that are strongly rectified (ΔeagD, ΔPAS-cap, and E600R cNBHD), the *V_m_* at which activation was maximal could not be accurately determined, and thus *V*_0.5_ and *k* values are likely to be underestimated. The time-dependent kinetics of activation were measured from calculations of time from 10 to 80% activation (*t*_10–80%_) using Tracan software (written in-house by Dr. Noel Davies). Data are presented as mean ± S.E. (*n* = number of cells). Statistical comparisons were performed using paired or unpaired Student's *t* tests where appropriate. Differences were considered significant at *p* ≤ 0.05. Figures and statistical analyses were prepared using Prizm software (GraphPad, San Diego).

##### Oocyte Calcium Imaging

For measurement of Ca^2+^*_i_*, oocytes were injected with 50 nl of 1 mm Oregon Green BAPTA-1 potassium salt (Invitrogen, United Kingdom) 1–3 h prior to fluorescence imaging on an Olympus IX81 microscope (Olympus, United Kingdom) equipped with an FV1000 confocal scanning unit. The indicator (estimated final concentration of 50 μm) was excited at 488 nm, and emission was collected at 500–600 nm via a 10 × 0.4 NA U-Plan-S-Apo lens. Images were acquired at a rate of 0.2 Hz with the confocal aperture set at maximum. To prevent cell movement during I and T application, a sealed blunt-ended glass micropipette was pressed gently on the top surface of the oocyte. The lens was focused 25–100 μm above the coverslip to detect the signal from a cross-section of the oocyte that had unrestricted access to I and T added to the bath. Fluorescence signals (*F*) were quantified using ImageJ (55) as the average value from three different peripheral regions of interest per oocyte (each >3500 μm^2^) after background signal subtraction. *F* was expressed relative to the pre-stimulus level (*F*_0_). Intracellular calibration of the Ca^2+^ levels reported by Oregon Green 488 BAPTA-1 proved unreliable. Instead, an extracellular calibration was carried out in a custom multiwell microchamber with a range of free Ca^2+^ levels set with EGTA/CaCl_2_/HEPES mixtures (Maxchelator, maxchelator.stanford.edu). This calibration was then used to convert the *F*/*F*_0_ increase induced by I and T to an approximate increase in Ca^2+^ using a *K_d_* of 170 nm and resting Ca^2+^*_i_* of 100 nm.

##### Molecular Modeling

Homology models of the hEAG PAS-cNBD complex were built using Modeler (56), with the mEAG1 structure (Protein Data Bank code 4LLO) (25) used as a template. The sequence identity for both domains is 98%. Surface conservation was calculated using Consurf (57). Sequence alignments were prepared using Uniprot and edited with ESPript (58), and molecular model illustrations were prepared with the PyMOL Molecular Graphics System, Version 1.8.0.2, Schrödinger, LLC.

## Author Contributions

J. S. M. designed the study, acquired the funding, assisted with data analysis, assisted with the calcium imaging experiments, and wrote the manuscript. E. L., M. H., and A. F. performed and analyzed the majority of the experiments. N. D. assisted with acquiring the funding and with analyzing and interpreting electrophysiological recordings. F. M. and P. J. S. contributed to molecular modeling and interpretation of structural implications of results. M. M.-S. designed, performed, and assisted with the analysis of the calcium imaging experiments in Fig. 2 and the writing of the manuscript. All authors approved the final version of the manuscript.

## Footnotes

2 The abbreviations used are:

KCNH: ether à go-go potassium channel family
hEAG1: human ether-à-go-go
hERG1: human ether-a-go-go-related gene
CaM: calmodulin
I and T: ionomycin and thapsigargin
PAS: Per Arnt Sim
PIP_2_: phosphoinositide 4,5 bisphosphate
cNBHD: cyclic nucleotide binding homology domain
eagD: eag domain
BD-N: BD-C1, BD-C2, CaM binding domains on the N (BD-N) and C (BD-C) termini
AP: action potential.
